# Evaluation of SARS-CoV-2 Serological Testing in Patients with Multiple Myeloma and Other Hematologic Malignancies on Monoclonal Antibody Therapies

**DOI:** 10.3390/diagnostics10120992

**Published:** 2020-11-24

**Authors:** Lenin Mahimainathan, Madhusudhanan Narasimhan, Rolando Corchado, Hetalkumari Patel, Ankit Kansagra, Sridevi Devaraj, Praveen Ramakrishnan Geethakumari, Alagarraju Muthukumar

**Affiliations:** 1 Department of Pathology, University of Texas Southwestern Medical Center, Dallas, TX 75390, USA; lenin.mahimainathan@utsouthwestern.edu (L.M.); madhusudhanan.narasimhan@utsouthwestern.edu (M.N.); 2 William P. Clements Jr. University Hospital (CUH), University of Texas Southwestern Medical Center, Dallas, TX 75235, USA; rolando.corchado@utsouthwestern.edu; 3 Harold C. Simmons Comprehensive Cancer Center, University of Texas Southwestern Medical Center, Dallas, TX 75390, USA; hetalkumari.patel@utsouthwestern.edu; 4 Department of Internal Medicine, Hematology/Oncology, University of Texas Southwestern Medical Center, Dallas, TX 75390, USA; ankit.kansagra@utsouthwestern.edu; 5 Department of Pathology and Immunology, Baylor College of Medicine, Houston, TX 77030, USA; 6 Division of Hematologic Malignancies and Stem Cell Transplantation, Harold. C. Simmons Comprehensive Cancer Center, University of Texas Southwestern Medical Center, Dallas, TX 75390, USA

**Keywords:** COVID-19, SARS-CoV-2, therapeutic monoclonal antibodies, serology, cross-reactivity, M-spike, hematological malignancy, multiple myeloma

## Abstract

Background: Patients with hematological malignancies (HM), including multiple myeloma (MM), frequently suffer from immune deficiency-associated infectious complications because of both the disease and the treatment. Alarming results from China and the UK confirm the vulnerability of HM patients to severe acute respiratory syndrome coronavirus 2 (SARS-CoV-2) infection-driven coronavirus disease 2019 (COVID-19). Given that the immunoassay interference from the endogenous monoclonal immunoglobulin (M paraprotein) and treatment antibodies continually challenges the MM management, it is critical to evaluate the SARS-CoV-2 serology tests for suspected interference/cross-reactivity. Methods: We compared the degree of interference in three SARS-CoV-2 serology assay platforms in HM patients with and without COVID-19 and on various therapeutic monoclonal antibody (t-mAb) treatments. Further, we confirmed the cross-reactivity in pooled samples from normal and COVID-19 + samples spiked with respective antibodies in vitro. Results: None of the 93 HM patient samples with or without t-MAbs showed cross-reactivity on any of the three serology platforms tested. Conclusions: The tested three serologic assays for SARS-CoV-2 are specific and do not have cross-reactivity with M-components or t-MAbs indicating that they can be used safely in oncology practice and in research exploring the immunologic response to COVID-19 in patients with HM.

## 1. Introduction

The impact of COVID-19 infection on patients with cancer remains to be elucidated. Initial reports have shown more severe disease and higher case-fatality rates for patients with cancer [1]. In a case series from Wuhan, among 13 patients with hematologic malignancies (HM), the case-fatality rate was 62% compared to 0% for a comparator group of healthcare providers with COVID-19 [2]. Another study from the UK comprising 35 HM patients, where 69% were receiving active therapy at the time of COVID-19 diagnosis, showed a significantly high mortality rate of 40%, implying that patients with HM are vulnerable to not only COVID-19 but also treatment complications [3].

Of relevance, owing to defective immune function and treatment-associated impairments, multiple myeloma (MM), the second most common HM, is at increased risk for infections relative to its immunocompetent counterparts [4,5,6]. Importantly, two independent studies have reported that COVID-19 infection contributes significantly to the mortality rate among the cohort of MM by 39% [7] and 54.6% [8]. Notably, this mortality rate for COVID-19 in the MM cohort is remarkably higher by about 36-52% than the case-fatality rate of 2.91% of the overall population. Thus, a timely and accurate diagnosis of SARS-CoV-2 infection is critical in patients with MM and other hematologic malignancies.

Polymerase chain reaction (PCR)-based viral detection is the current gold standard for determining SARS-CoV-2 infection. SARS-CoV-2 serology tests are useful in assessing the scale of recent infection in asymptomatic individuals, screening and identifying potential convalescent plasma donors for therapy, and evaluating vaccine efficacy during clinical trial and post-trial care [9]. A recent study has shown that the combination of rapid serology testing (immunoassay) along with nucleic acid testing significantly improves the diagnostic accuracy of COVID-19 infection.

Numerous constituents present in the biological samples can alter the accuracy of analyte quantification, thus erroneously elevating or lowering the signal or results. This continuous challenge of immunoassay interference can cause the misinterpretation of a patient’s results by the laboratory and major delays in intended therapy for the underlying malignancy. Therefore, the assessment of specificity and cross-reactivity of serology tests is an important step prior to their implementation for routine patient testing. At present, the evaluation of cross-reactivity in most of SARS-CoV-2 serology tests is limited to related human corona viruses and other respiratory viruses. It is known that monoclonal paraproteins present in MM patients interfere in several diagnostic tests [10]. Similarly, the interference of therapeutic monoclonal antibodies (t-MAb) present in the sera of patients, with HM, on diagnostic tests has been reported [11]. However, the potential cross-reactivity of M-proteins and t-MAbs on SARS-CoV-2 serology tests has not been studied. Thus, the investigation of suspected interference in SARS-CoV-2 serology testing will be crucial to prevent any inconsistencies that may occur between clinical and laboratory findings for proper clinical management of this population.

## 2. Materials and Methods

**Figure 1:**
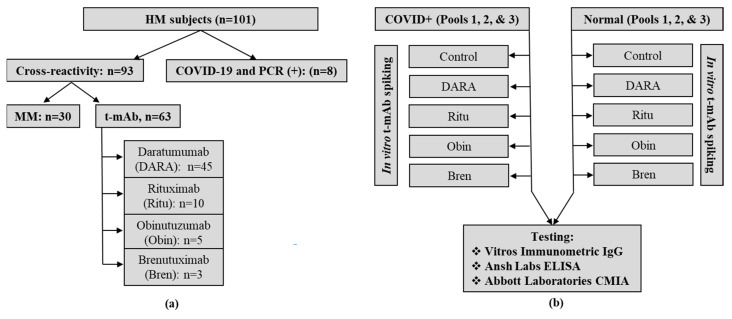
(**a**) Flowchart of severe acute respiratory syndrome coronavirus 2 (SARS-CoV-2) serology cross-reactivity testing study cases. Hundred and one unique hematological malignancy (HM) patient samples were included in SARS-CoV-2 serology testing. Eight of those were coronavirus disease 2019 (COVID-19)-positive by PCR, 30 were multiple myeloma (MM) patients and the remaining HM patients were on therapeutic monoclonal antibody (t-mAb) treatments. (**b**) Flowchart to assess the degree of interference in three SARS-CoV-2 serology assays due to different therapeutic monoclonal antibodies in normal and COVID-19 + samples by supplementing/spiking with respective antibodies in vitro. Pooled samples from normal and COVID-19 + patients were spiked with the clinically relevant concentration of each of the four therapeutic monoclonal antibodies—DARA (0.5 g/L), Ritu (0.5 g/L), Obin (0.5 g/L) and Bren (0.15 g/L)—or equal volume of saline for controls. These specimens then underwent analysis for the respective COVID-19 serology assays. Three pooled samples were used in each group (n = 3).

This is a retrospective study comprising 101 unique serum samples from HM and HM+CoV patients collected between March and October 2020, including samples from 30 MM patients with measurable M-spikes, 8 HM + CoV cases and the remaining cases (n = 63) from patients in various t-MAb treatments (Daratumumab n = 45, Rituximab n = 10, Obinutuzumab n = 5 and Brentuximab n = 3) (Figure 1a). All were remnant samples in the laboratory after routine testing. The UT Southwestern Medical Center’s Institutional Review Board (IRB) approved this study (STU-2020-0366; 04/17/2020).

Serologic assessment for SARS-CoV-2 was performed by Abbott Architect IgG CMIA (nucleocapsid), Ansh Labs IgG ELISA test on Dynex DSX (S1/S2) and Ortho Vitros immunometric total antibody (including IgA, IgM and IgG, S1) tests (Table S1). In addition, we spiked 4 t-MAbs at clinically relevant concentrations (0.15 to 0.5 g/L) or equal volume of saline for controls in three COVID-19-positive and three routine random-pooled samples (with no record of COVID-19 PCR positivity) and tested for SARS-CoV-2 serology to ascertain their reactivity (Figure 1b). M2000 Abbott Real-Time SARS-CoV-2 assay or the Abbott ID NOW COVID-19 assay confirmed infection with SARS-CoV-2 in the HM patients [12]. Cross reactivity was assessed in terms of positive or reactive results for the above tested assays in the non-COVID+HM specimens.

## 3. Results and Discussion

Our study encompassed a broad cross-section of hematologic malignancies and thus allowed us to assess the impact of various M-components and tMAbs on the three tested serology assay systems (Table S2). In addition, all the eight HM+CoV PCR+ patient samples showed positivity by all three antibody tests demonstrating their specificity. In this cohort, most patients had MM (6/8), four needed hospitalization and only one patient with relapsed/refractory acute lymphoblastic leukemia (ALL) died from complications of COVID-19 (Table S3). None of the 93 HM patient samples with or without t-MAbs showed cross-reactivity on any of the three serology platforms tested (Table 1a).

The various M-components did not impact the serological assays. We performed spiking studies with t-MAbs in COVID + specimens to assess whether antibody treatment interacts and cross-reacts with the SARS-CoV-2 serological assays (Table 1b; Control in the Pooled COVID-19 samples highlighted in blue). Supplementation of t-MAbs in the reaction did not show a drastic increase or decrease in the Abbott and Ansh Labs. However, we noted a ~45% deviation beyond the allowable error limit in one of the Rituximab samples tested by Vitros and a likely reason could be because of the matrix-based difference (Table 1b; #1 in the Pooled COVID-19 column highlighted in blue). Importantly, these t-MAbs did not cross-react with the tested SARS-CoV-2 serological assays in the pooled normal samples (Table 1b; column highlighted in green). Altogether, these data confirm the specificity of the tested platforms.

These qualitative assays, although have a limitation in that any value under the threshold (positive/reactive for SARS-CoV-2) is considered as non-reactive. But a value proximal to threshold may either denote a weak reactive sample (presumptive positive) or waning antibody levels or may indicate cross-reactivity that we have not observed in the present study. The use of quantitative serological assays would be desirable.

## 4. Conclusions

In conclusion, our results demonstrate that the tested three serologic assays for SARS-CoV-2 are highly specific and do not have cross-reactivity with M-components or t-MAbs. Given a third wave of COVID-19 threatening the world, these assays can be safely used in oncology practice and in research exploring the immunologic response to COVID-19 in patients with hematologic malignancies.

## Figures and Tables

**Table 1:** (**a**) SARS CoV-2 serology assay test results of hematological malignancy patients treated with and without anti-myeloma monoclonal antibodies or having suspected COVID-19 infection. (**b**) SARS CoV-2 serology test results of normal and COVID-19 positive pooled samples spiked with therapeutic anti-monoclonal antibodies in vitro.

**(a)**													
**Specimen**		**Vitros Immunodiagnostics**					**Ansh Labs**				**Abbott Laboratories**		
		**Result**			**Interpretation**		**Result**			**Interpretation**	**Result**		**Interpretation**
MM (n = 30) mean ± SD		0.030 ± 0.009			NR		2.107 ± 0.842			NR	0.030 ± 0.081		Neg
HM + t-mAb (n = 63) mean ± SD	DARA (n = 45)	0.026 ± 0.007			NR		2.393 ± 1.130			NR	0.028 ± 0.084		Neg
	Ritu (n = 10)	0.040 ± 0.012			NR		2.360 ± 1.313			NR	0.067 ± 0.156		Neg
	Obin (n = 5)	0.042 ± 0.019			NR		2.640 ± 1.165			NR	0.080 ± 0.140		Neg
	Bren (n = 3)	0.050 ± 0.010			NR		1.700 ± 0.721			NR	0.027 ± 0.015		Neg
HM + CoV (n = 8) Index or Unit (*)	Patient #1	10.2			R		10.2			INT	1.80		Pos
	Patient #2	211			R		211			R	5.65		Pos
	Patient #3	840			R		118.98			R	7.12		Pos
	Patient #4	770			R		95.55			R	7.32		Pos
	Patient #5	810			R		130.72			R	8.11		Pos
	Patient #6	940			R		132.42			R	7.62		Pos
	Patient #7	590			R		82.97			R	7.58		Pos
	Patient #8	620			R		34.71			R	7.05		Pos
**(b)**													
**Assay Type**		**Pooled Normal Samples—** **Index/Unit (Non-Reactive/Negative)**						**Pooled COVID-19 Samples** **—** **Index/Unit (Reactive/Positive)**					
		**Control**	**DARA**	**Ritu**	**Obin**	**Bren**		**Control**	**DARA**		**Ritu**	**Obin**	**Bren**
Vitros Immuno-diagnostics	1	0.02	0.02	0.02	0.02	0.02		135	154		196	137	158
	2	0.02	0.02	0.02	0.02	0.02		11.5	10.9		11.0	10.9	12.1
	3	0.02	0.02	0.02	0.02	0.02		460	442		448	448	437
Ansh Labs	1	1.6	1.3	1.1	2.1	3.1		98.80	96.79		85.04	100.16	81.20
	2	2.5	3.1	2.5	1.3	2.1		15.84	14.00		13.64	16.66	13.43
	3	2.4	3.1	1.5	3.6	4.1		95.16	101.68		97.32	105.14	94.67
Abbott Laboratories	1	0.02	0.02	0.02	0.02	0.02		6.89	6.89		7.15	6.89	7.03
	2	0.08	0.08	0.07	0.08	0.08		1.86	1.90		1.79	1.83	1.84
	3	0.07	0.07	0.07	0.07	0.08		6.35	6.24		6.45	6.41	6.33

(a) NR—non-reactive; R—reactive; INT—intermediate; Neg—negative; Pos—positive. The values presented are index/unit recommended by the corresponding manufacturer with interpretation of the results, non-reactive or negative indicating the specimens are negative for anti-SARS-CoV-2. In contrast, the results such as reactive, intermediate or positive are collectively interpreted to be positive specimens for anti-SARS-CoV-2. *The characteristics for the corresponding patient samples are provided in Table S3. (b) The following index/unit values < 1.0 and < 10.0 are non-reactive, and ≥ 1.0 and ≥ 12.0 are reactive in Vitros and Ansh Labs assay, respectively; value < 1.4 is negative and ≥ 1.4 is positive in Abbot Laboratories assay. Non-reactive or negative indicates the specimens are negative for anti-SARS-CoV-2 and, in contrast, reactive or positive indicates the specimens are positive for anti-SARS-CoV-2.

## Supplementary Materials

The following are available online at https://www.mdpi.com/2075-4418/10/12/992/s1, Table S1: Summary of the serology assays included, Table S2: Clinical characteristics of patients included in this study, Table S3: Clinical characteristics of patients with hematologic malignancies who were positive for SARS-CoV-2 infection by PCR.
