# Supplementary material for: Evaluation of SARS-CoV-2 Serological Testing in Patients with Multiple Myeloma and Other Hematologic Malignancies on Monoclonal Antibody Therapies
Source: Diagnostics (Basel). 2020 Nov 24;10(12):992. doi: 10.3390/diagnostics10120992 (PMC7760559; doi:10.3390/diagnostics10120992)
Supplement: Supplementary file 1 [file diagnostics-10-00992-s001.zip › diagnostics-1008668-supplementary.pptx]

## Slide 1
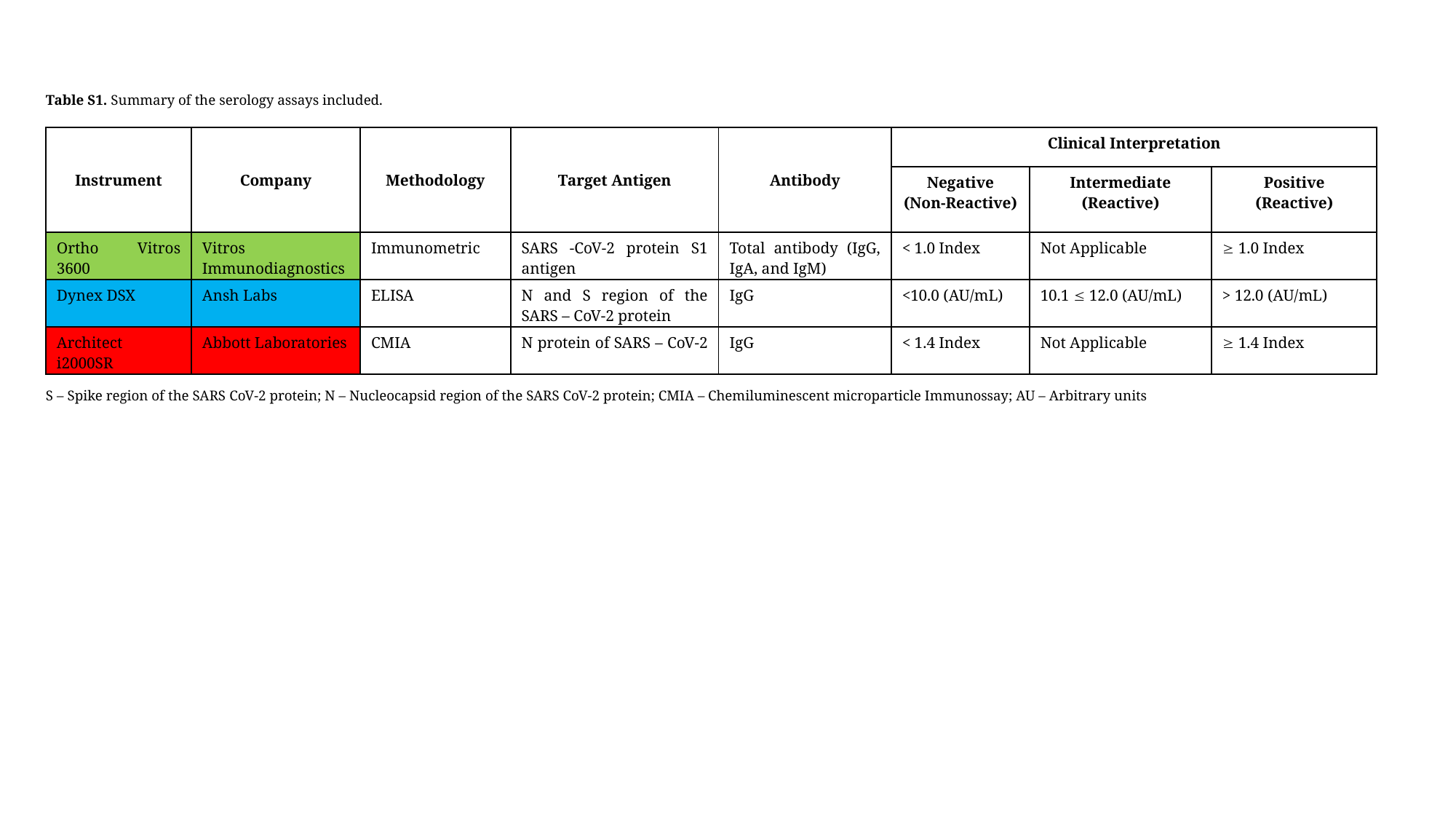

Table S1. Summary of the serology assays included.
| Instrument | Company | Methodology | Target Antigen | Antibody | Clinical Interpretation | | |
| --- | --- | --- | --- | --- | --- | --- | --- |
| | | | | | Negative (Non-Reactive) | Intermediate (Reactive) | Positive (Reactive) |
| Ortho Vitros 3600 | Vitros Immunodiagnostics | Immunometric | SARS -CoV-2 protein S1 antigen | Total antibody (IgG, IgA, and IgM) | < 1.0 Index | Not Applicable | ≥ 1.0 Index |
| Dynex DSX | Ansh Labs | ELISA | N and S region of the SARS – CoV-2 protein | IgG | <10.0 (AU/mL) | 10.1  12.0 (AU/mL) | > 12.0 (AU/mL) |
| Architect i2000SR | Abbott Laboratories | CMIA | N protein of SARS – CoV-2 | IgG | < 1.4 Index | Not Applicable | ≥ 1.4 Index |
S – Spike region of the SARS CoV-2 protein; N – Nucleocapsid region of the SARS CoV-2 protein; CMIA – Chemiluminescent microparticle Immunossay; AU – Arbitrary units

## Slide 2
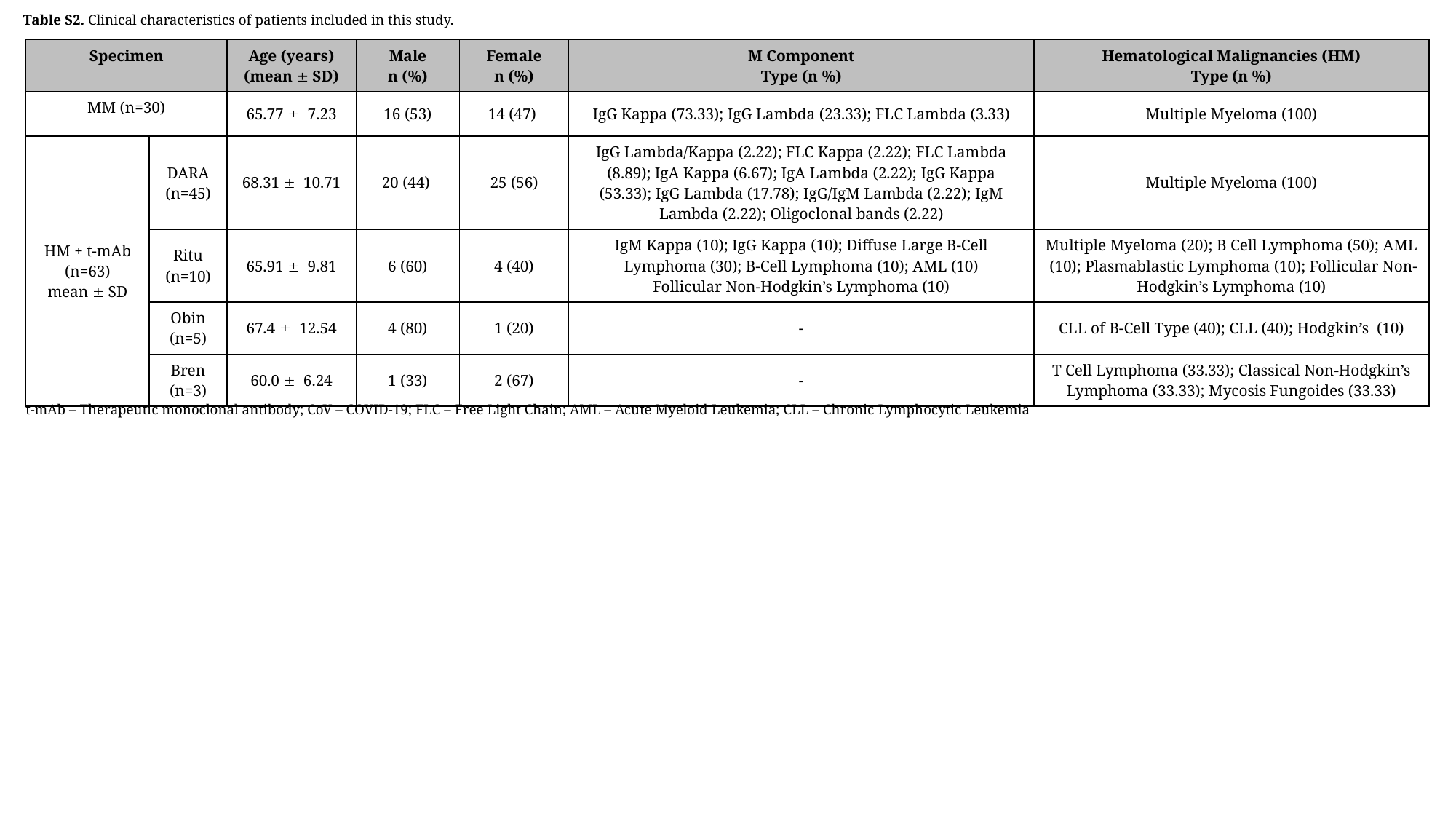

Table S2. Clinical characteristics of patients included in this study.
| Specimen | | Age (years) (mean ± SD) | Male n (%) | Female n (%) | M Component Type (n %) | Hematological Malignancies (HM) Type (n %) |
| --- | --- | --- | --- | --- | --- | --- |
| MM (n=30) | | 65.77 ± 7.23 | 16 (53) | 14 (47) | IgG Kappa (73.33); IgG Lambda (23.33); FLC Lambda (3.33) | Multiple Myeloma (100) |
| HM + t-mAb (n=63) mean ± SD | DARA (n=45) | 68.31 ± 10.71 | 20 (44) | 25 (56) | IgG Lambda/Kappa (2.22); FLC Kappa (2.22); FLC Lambda (8.89); IgA Kappa (6.67); IgA Lambda (2.22); IgG Kappa (53.33); IgG Lambda (17.78); IgG/IgM Lambda (2.22); IgM Lambda (2.22); Oligoclonal bands (2.22) | Multiple Myeloma (100) |
| | Ritu (n=10) | 65.91 ± 9.81 | 6 (60) | 4 (40) | IgM Kappa (10); IgG Kappa (10); Diffuse Large B-Cell Lymphoma (30); B-Cell Lymphoma (10); AML (10) Follicular Non-Hodgkin’s Lymphoma (10) | Multiple Myeloma (20); B Cell Lymphoma (50); AML (10); Plasmablastic Lymphoma (10); Follicular Non-Hodgkin’s Lymphoma (10) |
| | Obin (n=5) | 67.4 ± 12.54 | 4 (80) | 1 (20) | - | CLL of B-Cell Type (40); CLL (40); Hodgkin’s (10) |
| | Bren (n=3) | 60.0 ± 6.24 | 1 (33) | 2 (67) | - | T Cell Lymphoma (33.33); Classical Non-Hodgkin’s Lymphoma (33.33); Mycosis Fungoides (33.33) |
t-mAb – Therapeutic monoclonal antibody; CoV – COVID-19; FLC – Free Light Chain; AML – Acute Myeloid Leukemia; CLL – Chronic Lymphocytic Leukemia

## Slide 3
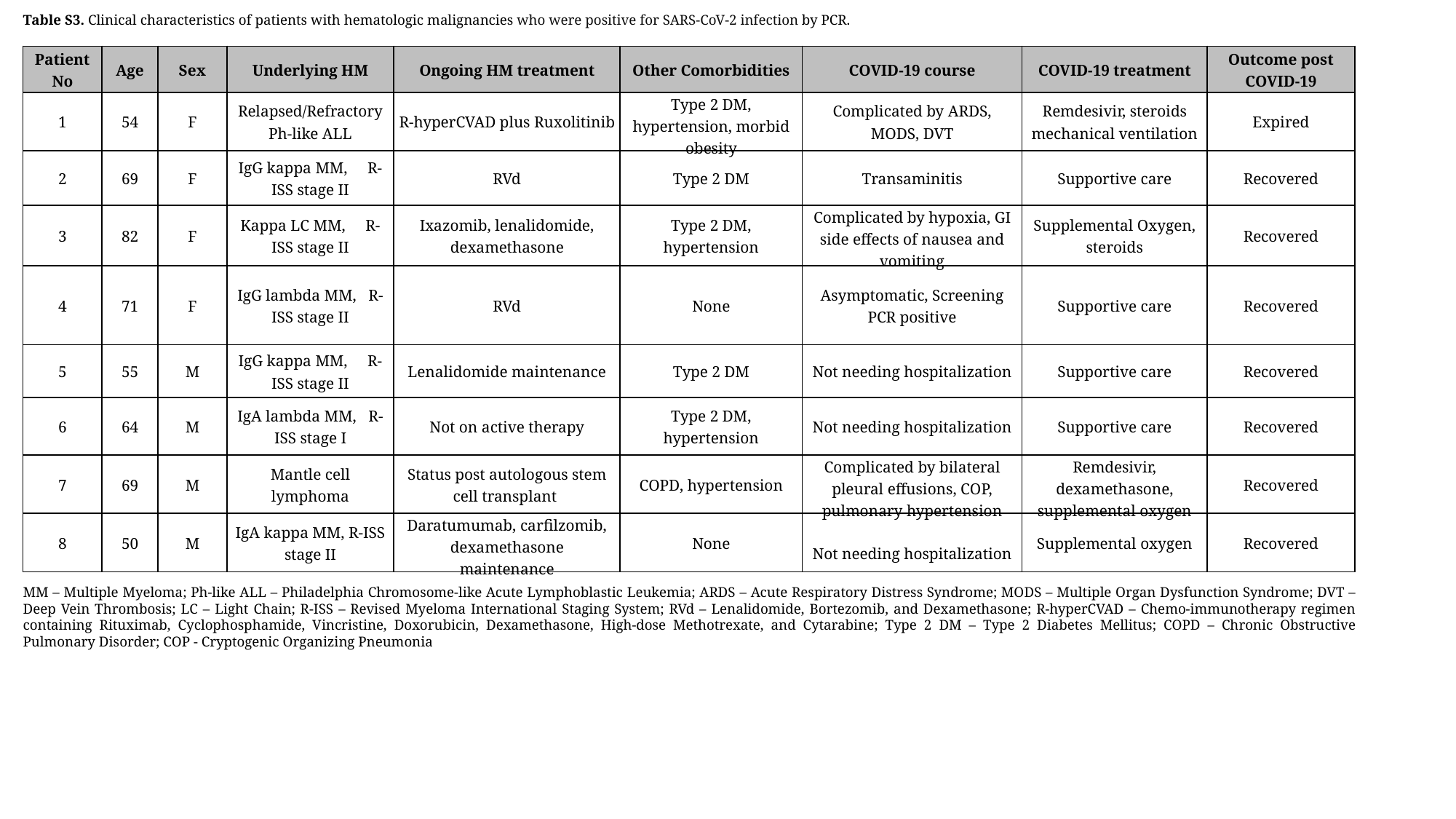

Table S3. Clinical characteristics of patients with hematologic malignancies who were positive for SARS-CoV-2 infection by PCR.
| Patient No | Age | Sex | Underlying HM | Ongoing HM treatment | Other Comorbidities | COVID-19 course | COVID-19 treatment | Outcome post COVID-19 |
| --- | --- | --- | --- | --- | --- | --- | --- | --- |
| 1 | 54 | F | Relapsed/Refractory Ph-like ALL | R-hyperCVAD plus Ruxolitinib | Type 2 DM, hypertension, morbid obesity | Complicated by ARDS, MODS, DVT | Remdesivir, steroids mechanical ventilation | Expired |
| 2 | 69 | F | IgG kappa MM, R-ISS stage II | RVd | Type 2 DM | Transaminitis | Supportive care | Recovered |
| 3 | 82 | F | Kappa LC MM, R-ISS stage II | Ixazomib, lenalidomide, dexamethasone | Type 2 DM, hypertension | Complicated by hypoxia, GI side effects of nausea and vomiting | Supplemental Oxygen, steroids | Recovered |
| 4 | 71 | F | IgG lambda MM, R-ISS stage II | RVd | None | Asymptomatic, Screening PCR positive | Supportive care | Recovered |
| 5 | 55 | M | IgG kappa MM, R-ISS stage II | Lenalidomide maintenance | Type 2 DM | Not needing hospitalization | Supportive care | Recovered |
| 6 | 64 | M | IgA lambda MM, R-ISS stage I | Not on active therapy | Type 2 DM, hypertension | Not needing hospitalization | Supportive care | Recovered |
| 7 | 69 | M | Mantle cell lymphoma | Status post autologous stem cell transplant | COPD, hypertension | Complicated by bilateral pleural effusions, COP, pulmonary hypertension | Remdesivir, dexamethasone, supplemental oxygen | Recovered |
| 8 | 50 | M | IgA kappa MM, R-ISS stage II | Daratumumab, carfilzomib, dexamethasone maintenance | None | Not needing hospitalization | Supplemental oxygen | Recovered |
MM – Multiple Myeloma; Ph-like ALL – Philadelphia Chromosome-like Acute Lymphoblastic Leukemia; ARDS – Acute Respiratory Distress Syndrome; MODS – Multiple Organ Dysfunction Syndrome; DVT – Deep Vein Thrombosis; LC – Light Chain; R-ISS – Revised Myeloma International Staging System; RVd – Lenalidomide, Bortezomib, and Dexamethasone; R-hyperCVAD – Chemo-immunotherapy regimen containing Rituximab, Cyclophosphamide, Vincristine, Doxorubicin, Dexamethasone, High-dose Methotrexate, and Cytarabine; Type 2 DM – Type 2 Diabetes Mellitus; COPD – Chronic Obstructive Pulmonary Disorder; COP - Cryptogenic Organizing Pneumonia
